# Silencing of *Thrips palmi UHRF1BP1* and *PFAS* Using Antisense Oligos Induces Mortality and Reduces Tospovirus Titer in Its Vector

**DOI:** 10.3390/pathogens11111319

**Published:** 2022-11-10

**Authors:** Priti, Sunil Kumar Mukherjee, Amalendu Ghosh

**Affiliations:** Insect Vector Laboratory, Advanced Centre for Plant Virology, Indian Agricultural Research Institute, New Delhi 110012, India

**Keywords:** ASOs, RNAi, melon thrips, GBNV, virus–vector relationship

## Abstract

*Thrips palmi* (Thysanoptera: Thripidae) is an important pest of vegetables, legumes, and ornamentals. In addition, it transmits several plant viruses. *T. palmi* genes associated with innate immunity, endocytosis-related pathways, and cuticular development are highly enriched in response to *Groundnut bud necrosis orthotospovirus* (GBNV, genus *Orthotospovirus*, family *Tospoviridae*) infection. As the previous transcriptomic study suggested the involvement of *T. palmi UHRF1BP1* and *PFAS* in GBNV infection, these two genes were targeted for silencing using antisense oligonucleotides (ASOs), and the effects on thrips’ fitness and virus acquisition were observed. Phosphorothioate modification of ASOs was carried out by replacing the nonbridging oxygen atom with a sulfur atom at the 3′ position to increase nuclease stability. The modified ASOs were delivered orally through an artificial diet. Exposure to ASOs reduced the target mRNA expression up to 2.70-fold optimally. Silencing of *T. palmi UHRF1BP1* and *PFAS* induced 93.33% mortality that further increased up to 100% with an increase in exposure. Silencing of *T. palmi UHRF1BP1* and *PFAS* also produced morphological deformities in the treated *T. palmi*. GBNV titer in *T. palmi* significantly declined post-exposure to ASOs. This is the first-ever report of silencing *T. palmi UHRF1BP1* and *PFAS* using modified ASO to induce mortality and impair virus transmission in *T. palmi*. *T. palmi UHRF1BP1* and *PFAS* would be novel genetic targets to manage thrips and restrict the spread of tospovirus.

## 1. Introduction

Thrips (order Thysanoptera) are important pests of food, fiber, and fodder crops worldwide. Besides direct damage caused by sucking plant sap, they transmit several plant viruses including tospoviruses, illarviruses, carmoviruses, machlomoviruses, and sobemoviruses [1,2,3,4]. Tospoviruses cause more than a billion dollars annual losses worldwide [5]. Tospovirus propagates within thrips; thus, thrips serve as an alternate host of tospovirus. Transmission of tospovirus by thrips is also unique. Thrips can only transmit the tospovirus in the adult stage if virus acquisition takes place during the early larval instar. Melon thrips, *Thrips palmi* is one of the predominant thrips species in Asian countries [6,7]. It infests almost all vegetables, legumes, and ornamentals. Cucumbers, aubergine, beans, melons, potatoes, and berries are severely affected by *T. palmi*. It transmits around seven tospoviruses [8]. *T. palmi*-transmitted *Groundnut bud necrosis orthotospovirus* (GBNV, genus *Orthotospovirus*, family *Tospoviridae*) causes more than USD 80 million losses annually in Asia [9]. GBNV can cause yield losses up to 100% in tomatoes [10,11]. Outbreaks of *Watermelon bud necrosis orthotospovirus* (WBNV) transmitted by *T. palmi* resulted in the failure of watermelon cultivation in southern India [12]. The knowledge of host plant resistance to thrips vectors as well as tospoviruses is very limited. Pesticides are commonly used to maintain a very low vector population and restrict the spread of tospoviruses. However, thrips develop resistance to insecticides quickly. Moreover, insecticides are hazardous to human health and the overall ecology. Identification of potential genetic targets to interrupt the thrips–tospovirus relationship is one of the facile approaches to the sustainable management of thrips–tospovirus.

*T. palmi* genes responsive to GBNV and capsicum chlorosis virus (CaCV) infection are partially known [13,14]. The response of about 103 genes of *T. palmi* is conserved following infection by GBNV and CaCV. *T. palmi* genes associated with innate immunity, endocytosis-related pathways, and cuticular development are highly enriched in response to GBNV infection [14]. GBNV probably enters the vector cells following clathrin-mediated endocytosis [15]. The genes encoding endocytosis-related pathways involving ubiquitin-like activity that binds the PHD and RING finger domains and named UHRF1BP1, nephrin, proteoglycan 4-like, and heparin sulfate proteoglycan (HSPG) were highly upregulated in viruliferous *T. palmi*. UHRF1BP1 is recruited in the early endosome resulting in aberrant tubulation of endosomal membranes [16]. Significant overexpression of *T. palmi* UHRF1BP1 in response to GBNV infection indicates its putative role in viral endocytosis and membrane fusion [14]. Genes like *phosphoribosylformylglycinamidine synthase* (*PFAS*), *heat shock protein 70* (*hsp70*), *sperm-associated antigen 6* (*spag6*)-like, and *laccase-5* are also overexpressed in viruliferous *T. palmi* as a part of the innate immune response [14]. In the current study, *T. palmi UHRF1BP1* and *PFAS* were silenced using antisense oligonucleotides (ASOs) to assess the effect on GBNV acquisition and vector fitness.

ASOs are short, synthetic, single-stranded deoxyribonucleotides complementary to the target mRNA that can silence the protein expression [17]. Silencing of a molecular target by ASOs is achieved by induction of RNase H endonuclease activity that cleaves the RNA component of the RNA–DNA heteroduplex. ASOs can also act through inhibition of 5′ cap formation, modulation of RNA splicing, modulation of polyadenylation, microRNA inhibition, and/or steric hindrance of ribosomal activity [18,19,20]. ASOs in their naive form are rapidly digested, thus limiting their bioavailability in the disease-associated tissues [21]. Several chemical modifications of the ASOs such as the inclusion of phosphorothioate, addition of 2′-O-Methyl and 2′-O-Methoxyethyl groups, and others led to the formation of peptide nucleic acid, locked nucleic acid, and phosphorodiamidate morpholino oligomer, etc. are known to enhance target affinity, specificity, nuclease resistance, bio-stability, and penetration to tissues [18]. The present work is the first experimental evidence of the efficacy of phosphorothioate-modified ASOs in silencing the mRNA expression of thrips. The study also demonstrates the proof of concept for the potentiality of the putative gene targets in the management of thrips and tospovirus.

## 2. Materials and Methods

### 2.1. T. palmi Population

An isofemale line of *T. palmi*, generated from a single female was used in this study. The population was maintained on aubergines (*Solanum melongena* var. Navkiran, Mahyco Pvt. Ltd., Delhi, India) since 2018 under controlled conditions at 28 ± 1 °C temperature, 60 ± 10% relative humidity, and 16 h light and 8 h dark photoperiod. The identity of the population was substantiated by morphological keys and sequencing mitochondrial cytochrome oxidase subunit I.

### 2.2. Virus Culture

The GBNV isolate (GenBank accession no. MN566913 [14]) maintained at IARI, New Delhi was used in the study. Healthy cowpea plants (*Vigna unguiculata* var. Pusa Komal) were sap-inoculated with GBNV, as described by Ghosh et al. [22]. The GBNV-infected plants were maintained at 25 ± 1 °C, 60 ± 10% relative humidity, and 16 h of light and 8 h of dark under insect-proof conditions and tested in reverse-transcriptase PCR (RT-PCR) using GBNV-specific primers, AG109F and AG110R [14].

### 2.3. Designing and Synthesis of Antisense Oligonucleotides

**Figure 1:**
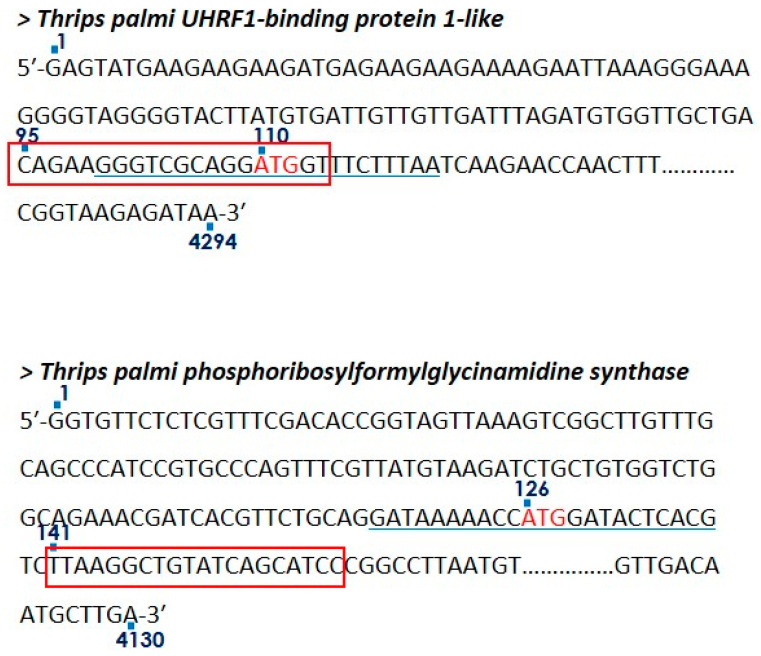
Target regions of ASOsin *T. palmi UHRF1BP1* and *PFAS*. “ATG” is delineated in red letters. The underlines indicate the Kozak sequences. Complementary target regions of ASOs are indicated in the box.

Genes were selected from upregulated transcripts of *T. palmi* in response to GBNV infection [14]. Among the upregulated transcripts, *T. palmi UHRF1BP1* and *PFAS* were abundant in viruliferous *T. palmi* and were selected in the current study to silence using ASOs. ASOs were designed using Sfold software (https://sfold.wadsworth.org/cgi-bin/soligo.pl (accessed on 28 July 2021) for statistical folding and regulatory RNAs. Stairwell for designing ASOs went through the following steps: (a) prediction of secondary structures with minimal free energy; (b) a local secondary structure identification for accessibility to ASOs which are usually located at the terminal end, internal loops, joint sequences, hairpins, and bulges of 10 or more consecutive nucleotides; (c) absence of GGGG, as it weakens the ASO activity; (d) optimal 40–60% GC content; and (e) binding energy (∆G°37) of mRNA and ASO should be ≥−8 kcal/mol. Kozak sequences of transcripts were preferred as target sites for ASO designing as blocking this nucleic acid motif produces a strong impairment of mRNA recognition and binding by ribosomal protein. Nucleotides at +4, −3, and −6 positions in the Kozak sequence are important in the initiation of translation and strong consensus [23,24,25], where the A nucleotide of the “AUG” is delineated as +1 in mRNA sequences. The design of ASOs has been shown in Figure 1. Phosphorothioate modification was undertaken in the DNA backbone by replacing the nonbridging oxygen atom with a sulfur atom at the 3′ position to enhance nuclease stability. The phosphorothioate modifications were done at terminal ends and +4, −3, and −6 nucleotide positions of the Kozak sequence in the ASO. Phosphorothioate-modified ASOs were synthesized by Novel Gene (Hyderabad, India). The list of ASOs used in the present study is included in Table 1.

### 2.4. Delivery of ASOs

**Figure 2:**
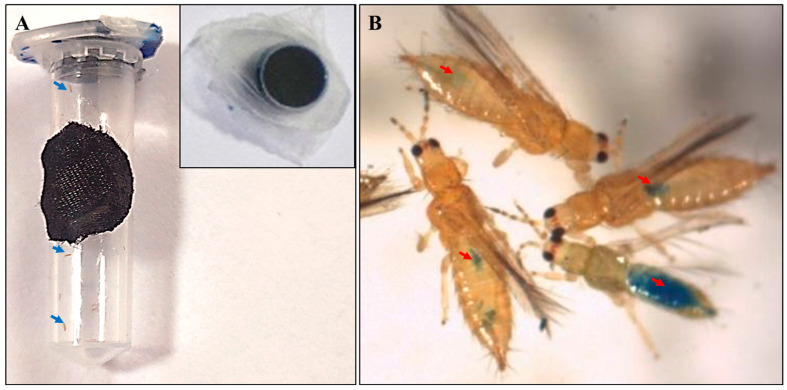
Setup for delivery of ASOs. (**A**) A microcentrifuge tube was modified to deliver the ASOs mixed with an artificial diet. The cap of the tube was filled with their diet and covered with stretched Parafilm M membrane. The adults of *T. palmi* within the microcentrifuge tube are indicated by red arrows. (**B**) *T. palmi* adults fed on the artificial diet mixed with blue tracker dye. The feeding was confirmed by the presence of a blue tinge on the abdomen indicated by red arrows.

ASOs were orally delivered to *T. palmi* adults and larvae mixed with an artificial diet. An artificial feeding setup and delivery of ASOs to *T. palmi* were optimized (Figure 2A). In our previous study, pine pollen with 10% honey solution recorded a maximum fitness of *T. palmi* [26]. In the current study, pine pollen was preferred over the LB diet [27] and TSBY diet [28] based on the survivability of *T. palmi* (data not shown). An extract of pine pollen was prepared by boiling 25 mg/mL of pine pollen (Lost Empire Herbs, Watsonville, CA, USA) at 70 °C for 20 min with intermittent vortex. The suspension was allowed to precipitate for 30 min. The upper aqueous phase was taken out and 1% sucrose (Himedia, Mumbai, India) was added to prepare the diet. A 0.0001% methylene blue tracker dye (Bio Basic, Amherst, MA, USA) was mixed with the diet. The methylene blue tracker dye is non-toxic to thrips [27] and was visible through the thrips cuticles, thus helping distinguish the fed thrips from non-fed thrips. The aqueous diet with methylene blue dye was filter-sterilized before use.

The caps of 2 mL microcentrifuge tubes were filled with 300 µL of diet mixed with dye and covered with a tightly stretched UV-treated Parafilm M membrane (Figure 2A). A small hole was made on the side wall of the microcentrifuge tubes and covered with a black cotton cloth for good aeration. *T. palmi* adults or larvae were released inside the tubes and the tubes were closed with diet-filled caps. The whole process was performed under sterile conditions in a laminar flow hood. The setups with thrips were kept at 28 ± 1 °C temperature, 60 ± 10% relative humidity, and 16 h light–8 h dark photoperiod.

### 2.5. Efficacy of ASOs on Survival of Thrips palmi Adults

ASOs were delivered to *T. palmi* adults, as described above. The initial dose–response of ASOs was carried out by taking 100, 600, and 1200 pmol/mL. Based on the initial dose–response results, ASOs were mixed with the artificial diet at a final concentration of 600 pmol/mL. Ten *T. palmi* adults were released in each tube in three biological replicates and allowed to feed constantly. An artificial diet without ASO and a diet mixed with respective sense oligos were taken as controls. The actively fed thrips confirmed by the presence of a blue tinge in the abdomen were only considered in the assay. The mortality of treated thrips was recorded at 1-, 4-, and 8-days post-application (DPA). The whole experiment was repeated three times. The mean mortality in ASO-fed thrips was calculated and compared with the thrips fed on the diet without ASO and the diet with sense oligo. Mean differences among the categories were separated by Tukey’s test at a confidence interval of 95% using XLSTAT 2014.5.03.

*T. palmi* adults exposed to ASO were checked under a stereo microscope (Stemi 2000c, Zeiss, Jena, Germany) for any induced morpho-deformities. The surviving *T. palmi* from several such replicates were used for determining the relative expression of target mRNAs, as described below. AG459-UHRF1BP1-ASO and AG461-PFAS-ASO were also fed to *Bemisia tabaci* (Hemiptera: Aleyrodidae) adults mixed with an artificial diet and blue tracker dye, as described by Chakraborty and Ghosh [29] to assess if there was any effect on a non-target organism.

### 2.6. Estimating Relative Expression of T. palmi UHRF1BP1 and PFAS mRNA

The relative expression of *T. palmi UHRF1BP1* and *PFAS* post-ASO exposure was estimated in RT-qPCR. *β-tubulin* was considered an endogenous control. The surviving *T. palmi* adults at 1, 4, and 8 DPA were collected using a fine Camel hairbrush. Five adults from each of the three different sets of biological replicates per treatment were used. Total RNA was isolated from *T. palmi* adults using the NucleoSpin RNA XS kit (Macherey-Nagel, Düren, Germany) following the manufacturer’s protocol. cDNA was synthesized using RevertAid First Strand cDNA Synthesis Kit (Thermo Fisher Scientific, Waltham, MA, USA) in a thermocycler (T100, Bio-Rad, San Francisco, CA, USA) at 42 °C for 60 min followed by enzyme inactivation at 70 °C for 5 min. The reaction mixture contained 1X RT reaction buffer, 25 ng template RNA, 5 μM oligo dT primer, 1 mM dNTP mix, 10 U of RevertAid RT, and 1 U of RibLock RNase inhibitor. The relative qPCR assay was carried out in an Insta Q48M real-time PCR (Himedia) with the final volume of 20 μL reaction mixture containing 10 μL of 1X GoTaq qPCR Master Mix (Promega, Madison, WI, USA), 300 nM CRX reference dye, 0.25 μM each forward and reverse primer (AG369F-AG370R for *PFAS*, AG379F-AG380R for *UHRF1BP1*, and AG171F-AG172R for *β-tubulin*) (Table 2), and 2 μL template cDNA. Thermal cycling was performed as initial denaturation at 94 °C for 5 min, 30 cycles of 94 °C for 30 s, 56 °C for 30 s, and 72 °C for 30 s. Since BRYT Green dye binds non-specifically to any double-stranded DNA, a dissociation or melting stage was carried out after every reaction to determine the specificity of the amplicons based on the melting curve. The RT-qPCR was performed with two technical replicates for each of the three different sets of biological replicates. The fold change in expression was normalized by excluding the changes in the cycle threshold (C_T_) value of the endogenous control, *β-tubulin*. Log_2_ fold change value was calculated, and the relative expression of mRNA was determined by normalizing the log_2_ 2^−ΔΔCT^ values of the ASO-treated *T. palmi* with *T. palmi* fed on diet without ASO [30]. Mean differences among the categories were separated by Tukey’s test at a confidence interval of 95% using XLSTAT 2014.5.03. Statistical analysis and preparation of graphs were carried out in Microsoft Excel 2016.

### 2.7. Quantification of GBNV Copies in ASO-Fed T. palmi

*T. palmi* becomes viruliferous in the adult stage if tospovirus acquisition takes place during the early instar larval stage [22]. Thus, the effect of ASO on GBNV acquisition in thrips was assessed at the larval stage. Freshly emerged first instar larvae were collected from aubergine and fed to ASO following the above-mentioned protocol. A diet without ASO and a diet with sense oligo were considered as the control. After 2 h of feeding, the larvae were shifted to a GBNV-infected detached cowpea (*Vigna unguiculata* var. Pusa Komal) leaf in a virus acquisition setup, as described by Ghosh et al. [22]. The larvae were shifted to healthy detached aubergine leaves after 24 h of virus acquisition in insect breeding dishes and reared up to the adult stage under controlled conditions. Virus copies were estimated in the larvae (L2) post 48 h of virus acquisition and in freshly emerged adults. The surviving *T. palmi* from several replicates were used to maintain ten larvae in three biological replicates for each treatment. Total RNA was isolated, and cDNA was synthesized using RevertAid First Strand cDNA Synthesis Kit (Thermo Fisher Scientific) with an initial incubation at 25 °C for 5 min followed by 42 °C for 60 min and enzyme inactivation at 70 °C for 5 min. The reaction mixture contained 1X RT reaction buffer, 25 ng template RNA, 5 μM random hexamer primer, 1 mM dNTP mix, 10 U of RevertAid RT, and 1 U of RiboLock RNase inhibitor. The absolute quantification of the virus titer was performed in a qPCR. A 20 μL qPCR reaction mixture contained 10 μL of 1X GoTaq qPCR Master Mix (Promega), 300 nM CRX reference dye, 0.25 μM each forward and reverse primer (AG335F-AG336R, Table 2), and 2 μL template cDNA. Thermal cycling was performed as initial denaturation at 95 °C for 5 min, 35 cycles at 95 °C for 25 s, 54 °C for 25 s, and 72 °C for 30 s. Since BRYT Green dye binds non-specifically to any double-stranded DNA, a dissociation or melting stage was carried out after every reaction to determine the specificity of the amplicons. The qPCR was performed with two technical replicates for each of the three different biological replicates.

To prepare a standard curve of GBNV, the RT-PCR amplified product of GBNV-nucleocapsid protein (N) gene using primer pair AG335F-AG336R was ligated into pGEM-T easy vector system kit (Promega) and transformed into DH5α *E*. *coli* cells. A ten-fold serial dilution of the plasmid DNA from 20 ng to 2 × 10^−5^ ng was subjected to qPCR. The qPCR reaction was carried out in 20 μL of volume, as described above, taking 1 μL of serially diluted plasmid DNA as templates. To determine the assay reproducibility each dilution of the plasmid DNA was replicated thrice for constructing the standard curve of GBNV. The standard curve of GBNV was prepared by plotting a linear regression line with the mean C_T_ values on Y-axis and log_10_ DNA concentration on X-axis.

The absolute quantification of GBNV ingested by individual *T. palmi* larvae and adults was carried out by fitting the mean C_T_ values in the standard curve. The virus titer calculated in ng by extrapolating the mean C_T_ values in the standard curve was converted into the copy number using the formula N = (X ng*6.0221 × 10^23^ molecules/mole)/(n*340 g/mole*10^9^ ng/g), where N is the number of virus copies; X is the amount of amplicon in ng, and n is base pairs of recombinant plasmids. The average copy number of GBNV in individual *T. palmi* was calculated and compared among the ASO-exposed and non-exposed populations. The relative expression of *T. palmi UHRF1BP1* and *PFAS* at these time points was also estimated, as described above.

## 3. Results

### 3.1. Thrips palmi Population and Virus Culture

Adult males and females of the homogenous population of *T. palmi* were yellow in color. Males were comparatively smaller in size than female adults. The apex of the abdomen was blunt in males, whereas it had a sharp ovipositor in females. The head was quadrangular in shape with seven segmented antennae and the terminal segments were dark brown. There were three brick-red ocelli on the top of the head in a triangular fashion. One pair of interocellar setae originated outside the ocellar triangle.

A 653 bp nucleotide sequence of mitochondrial cytochrome oxidase subunit I was 100% identical to that of other *T. palmi* isolates (MN594549, MW020346) available in GenBank. The sequence can be retrieved with Accession No. MT992047.

The sap-inoculated leaves produced necrotic rings on the systemic leaves 10–14 days post-inoculation. RT-PCR with GBNV-specific primers produced an amplicon of 1.76 kb of the GBNV M segment. The nucleotide sequence of the amplified product showed more than 97% identity to GBNV isolates. The sequence can be retrieved from GenBank using Accession No. MN566913.

### 3.2. Design of Phosphorothioate-Modified ASO

Sfold showed the top 20 putative ASOs for *T. palmi UHRF1BP1* and *PFAS* mRNAs. AG459-UHRF1BP1-ASO was 20 nucleotides in length spanning 95–114 nucleotides of *UHRF1BP1* mRNA (Figure 1). It covered the Kozak sequence (100–122 nucleotide) of *T. palmi UHRF1BP1.* The GC content was 55%. No secondary structure was found on the target mRNA sequence as indicated by the unpaired probability of target site nucleotide (0.570) and ASO binding energy was −12.3 kcal/mol. AG461-PFAS-ASO was designed to target the 141–160 nucleotide position of *T. palmi PFAS* mRNA. Although Kozak sequences were preferred in designing the ASO, no putative ASO sequence spanning the Kozak sequence of *PFAS* (116–138 nucleotide) was found. The GC content of AG461-PFAS-ASO was calculated as 45%. No local secondary structures, internal loops, hairpins, and/or nucleotide bulges were predicted. The unpaired probability of the target site nucleotide was 0.563 and ASO binding energy was −8.9 kcal/mol. Both the ASOs were free from any ‘GGGG’ motif.

### 3.3. Effect of ASOs on the Relative Expression of T. palmi UHRF1BP1 and PFAS

*T. palmi* started feeding on the diet mixed with blue tracker dye within a few hours of release. The feeding was confirmed by the presence of a blue tinge on the abdomen (Figure 2B). The thrips which actively fed on the diet were only considered in the assay. An initial dose–response showed a steady increase in mortality of *T. palmi* at 600 pmol/mL compared to 100 pmol/mL of ASO feeding (Supplementary Figure S1). The mortality did not increase much with a further increase in ASO concentration (1200 pmol/mL). Therefore, ASOs at 600 pmol/mL were used throughout the study to evaluate their efficacy in silencing thrips genes, their effects on thrips fitness, and virus transmission.

**Figure 3:**
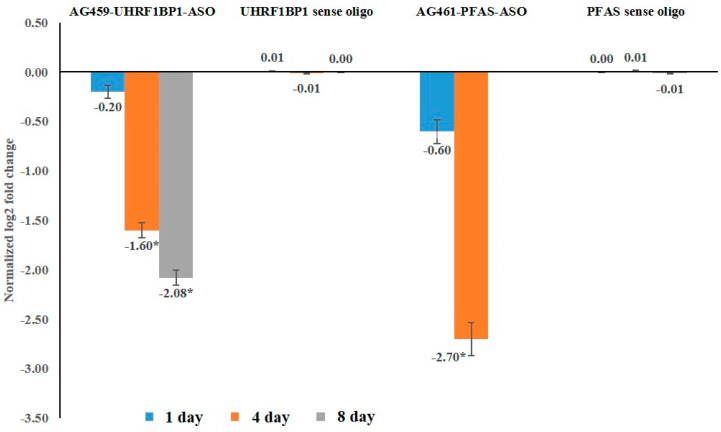
Expression of *T. palmi UHRF1BP1* and *PFAS* post-exposure to ASOs. The fold change in expression was normalized with the values in samples treated with the diet without ASO. As there was 100% mortality of *T. palmi* at 8 DPA of AG461-PFAS-ASO treatment, the expression of *PFAS* in surviving thrips could not be estimated. Mean differences among the categories were separated by Tukey’s test at a confidence interval of 95% using XLSTAT 2014.5.03. Mean denoted by an asterisk (*) indicates a significant difference (*p* < 0.05).

There was no significant (*p* = 0.821) regulation of target mRNA immediately after ASO exposure with respect to an endogenous control gene, *β-tubulin*. The expression of *UHRF1BP1* was significantly (*p* = 0.007) downregulated by 1.6-fold (Figure 3) in adult *T. palmi* at 4 DPA of AG459-UHRF1BP1-ASO feeding and it was further reduced by 2.08-fold at 8 DPA. Feeding on AG461-PFAS-ASO induced 2.70-fold downregulation of *T. palmi PFAS* at 4 DPA with respect to *T. palmi* fed on the diet without ASO. As there was 100% mortality of *T. palmi* at 8 DPA of AG461-PFAS-ASO treatment, the expression of *PFAS* could not be estimated. The reduction in mRNA expression level was significantly higher in AG461-PFAS-ASO treatment compared to AG459-UHRF1BP1-ASO. However, there was no significant regulation of *UHRPF1BP1* and *PFAS* in *T. palmi* adults fed on respective sense oligos up to 8 DPA indicating that the regulation in gene expression was specific to ASO exposure. The specific peaks of *UHRF1BP1*, *PFAS*, and *β-tubulin* amplicons at 83, 82, and 80 °C were not associated with any secondary peaks in the qPCR melting curve analysis indicating the specificity of the reactions.

### 3.4. Effect of Modified ASOs on Thrips Survival

**Figure 4:**
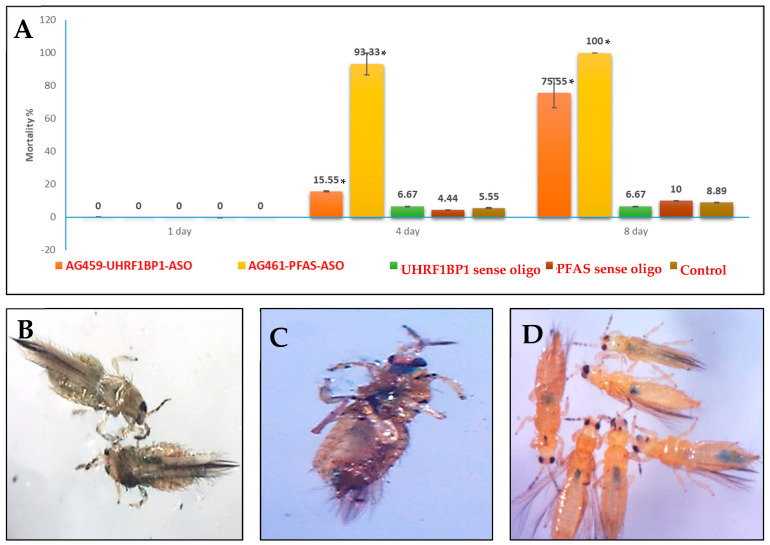
Effect of *T. palmi UHRF1BP1* and *PFAS* silencing on its survival. (**A**) Mortality of *T. palmi* post silencing *T. palmi UHRF1BP1* and *PFAS*. Mean differences among the categories were separated by Tukey’s test at a confidence interval of 95% using XLSTAT 2014.5.03. Mean denoted by an asterisk (*) indicates a significant difference (*p* < 0.05). (**B**) Silencing *T. palmi* UHRF1BP1 induced morpho-deformities in *T. palmi*. *T. palmi* adults exposed to AG459-UHRF1BP1-ASO became very flaccid, and the abdomen of the ASO-exposed thrips flattened. (**C**) In the case of AG461-PFAS-ASO, the exposed adult thrips became very brittle, squeezed and the appendages twisted. (**D**) No such effects were recorded in *T. palmi* adults fed on a diet without ASO and a diet mixed with the sense oligos.

There was no immediate effect of ASOs on the survival of *T. palmi* at 1 DPA. Exposure to AG459-UHRF1BP1-ASO induced 15.55% mortality in *T. palmi* at 4 DPA (Figure 4A). The mortality significantly (*p* = 0.017) increased up to 75.55% post 8 days of ASO feeding. The effect of AG461-PFAS-ASO on the survival of *T. palmi* adults was comparatively higher than AG459-UHRF1BP1-ASO. AG461-PFAS-ASO induced significantly (*p* = 0.038) higher mortality (93.33%) in *T. palmi* at 4 DPA. *T. palmi* adults exposed to AG461-PFAS-ASO did not survive up to 8 DPA, whereas the mean mortality of *T. palmi* adults fed on a diet without ASO ranged below 8.89%. There was no significant effect of UHRFBP1 and PFAS sense oligos on the survival of *T. palmi* adults that confirmed the mortality of *T. palmi* was specific to ASO treatment.

Remarkably, both the ASOs produced morpho-deformities in exposed *T. palmi* adults. *T. palmi* adults exposed to AG459-UHRF1BP1-ASO became very flaccid, and the abdomen of the ASO-exposed thrips flattened (Figure 4B). In the case of AG461-PFAS-ASO, the exposed adult thrips became very brittle, squeezed and the appendages twisted (Figure 4C). Whereas no such effects were recorded in *T. palmi* adults fed on a diet without ASO and a diet mixed with the sense oligos (Figure 4D). The ASOs targeting *T. palmi* mRNAs were also fed to *B. tabaci* to check if there was any effect on non-target organisms. No significant effect of AG459-UHRF1BP1-ASO and AG461-PFAS-ASO on *B. tabaci* fitness was recorded (Supplementary Figure S2).

### 3.5. Effect of ASOs on Virus Acquisition by T. palmi

**Figure 5:**
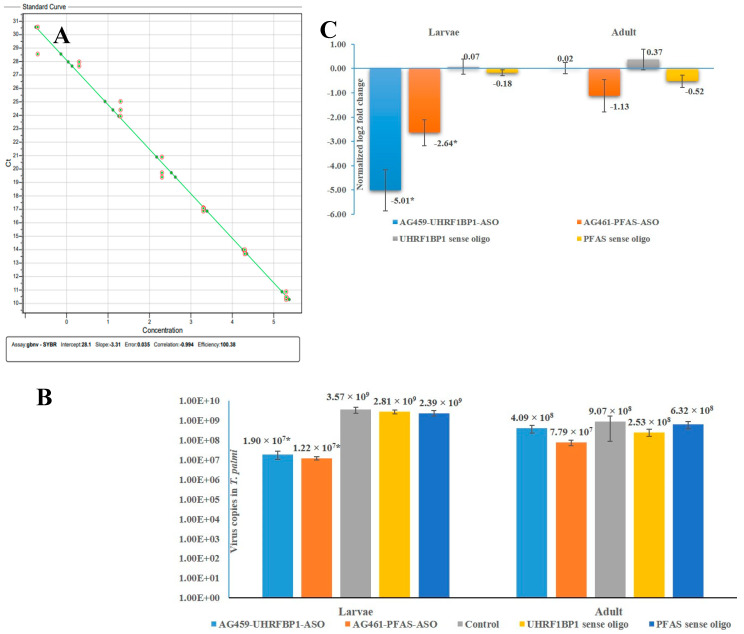
Effect of *T. palmi UHRF1BP1* and *PFAS* silencing on the acquisition of GBNV. (**A**) Standard curve of GBNV shows a linear relationship between log template concentrations in ng on the X-axis and C_T_ values on the Y-axis. Efficiency: 100.38%, correlation: −0.994. (**B**) Mean GBNV copies in *T. palmi* during L2 stage. (**C**) Expression of *T. palmi UHRF1BP1* and *PFAS* during L2 and adult stages post-exposure to ASOs in the L1 stage. The error bars are standard error of the mean (SEM). Mean denoted by an asterisk (*) indicates a significant difference (*p* < 0.05).

Primer pair AG335F-AG336R amplified a 219 bp fragment of GBNV-N gene in RT-PCR. The standard curve prepared using 10-fold dilutions of the cloned product in the pGEM-T vector showed a coefficient of correlation (R^2^) of −0.994 with a high amplification efficiency of 100.38%, indicating optimal conditions for absolute quantification (Figure 5A). In the melting curve analysis of qPCR, a single peak at 80 °C confirmed the specificity of the qPCR reactions.

The virus copies acquired by ASO-fed *T. palmi* were significantly less than the *T. palmi* fed on the diet without ASO. When larvae were not exposed to ASO, the GBNV copy number was 3.57 × 10^9^ in the L2 stage of *T. palmi* (Figure 5B), whereas the virus copy number was reduced post-exposure to ASOs. The reduction of virus copies was greater in AG461-PFAS-ASO treatment than in AG459-UHRF1BP1-ASO-treated *T. palmi*. The GBNV copies were significantly (*p* = 0.037) dropped (1.90 × 10^7^ copies) in L2 when exposed to AG459-UHRF1BP1-ASO. The relative expression of *T. palmi UHRF1BP1* was downregulated by 5.01-fold at this point (Figure 5C). Exposure to AG461-PFAS-ASO during L1 induced a 292.62-fold reduction (1.22 × 10^7^) of GBNV copies at the L2 stage. In corroboration with the virus copy number reduction, a 2.64-fold down-regulation of *T. palmi* PFAS expression was recorded at the L2 stage of AG461-PFAS-ASO-fed thrips. The *T. palmi* fed with sense oligos did not show any significant difference in GBNV copies compared to *T. palmi* fed on the diet without ASO, confirming that the effect on virus copies was specific to ASO treatment.

A portion of the larvae was allowed to grow up to the adults and GBNV copies were again quantified in the adult stage. The GBNV copies increased in the adult stage compared to L2 indicating propagation of GBNV in *T. palmi*. There was no significant difference (*p* = 2.82) in GBNV copies in the adult stage of both AG459-UHRF1BP1-ASO (4.09 × 10^8^ copies) and AG461-PFAS-ASO-exposed *T. palmi* (7.79 × 10^7^ copies) compared to *T. palmi* fed on the diet without ASO. After exposure to ASOs at the L1 stage, the expression of *T. palmi UHRF1BP1* and *PFAS* at the adult stage was also at par with *T. palmi* fed on diet without ASO. *T. palmi* molted into adults 8–10 days after ASO exposure. The effect of ASO might not be active across the molts and for a longer period.

## 4. Discussion

*T. palmi* is the predominant thrips species in the Asia-Pacific region. It was thought to be restricted in South East Asia until the 1980s. It has spread to Asia, Africa, Australia, North and South America, and the Caribbeans recently [6]. *T. palmi* has been listed as a quarantine pest by EPPO [31]. It causes 20–30% losses in vegetables and legumes. In addition, *T. palmi*-transmitted GBNV, WBNV, CaCV, and WSMoV cause significant yield losses in cucurbitaceous, solanaceous, and fabaceous plants [8,32].

The current study validates the functional role of *T. palmi UHRF1BP1* and *PFAS* in thrips fitness and GBNV transmission by gene silencing. *T. palmi UHRF1BP1* and *PFAS* were targeted based on our previous study that reported the upregulation of innate immune, endocytotic pathways, and cuticle development-associated genes in viruliferous *T. palmi* [14]. Among the upregulated transcripts, *T. palmi UHRF1BP1* and *PFAS* were abundant. Silencing of *T. palmi UHRF1BP1* and *PFAS* was achieved by oral administration of ASOs. ASO is successful in modulating gene expression with applications in drug discovery, functional genomics, and therapeutics [33,34,35,36,37]. This is the first evidence where silencing thrips genes has been achieved using ASO. ASO acts through multiple approaches, and thus is more successful to achieve target manipulation. However, ASO has an inherent limitation of nuclease instability. Several chemical modifications such as phosphorothioate, 2′-*O*-methyl, 2′-*O*-methoxyethyl, peptide nucleic acid, locked nucleic acid, and phosphorodiamidate morpholino oligomer have been adopted to enhance nuclease stability and efficacy [38]. In the current study, phosphorothioate modification was carried out at the terminal ends of the ASOs. Kozak sequence was generally preferred to design ASO due to its functional role in the initiation of translation. Nucleotides at the +4, −3, and −6 also underwent phosphorothioate modifications as they play an important role in translation initiation and consensus. However, no potential ASOs were found in *PFAS* mRNA that spanned the Kozak sequence. Hence, the best ASO based on the secondary structure, binding energy, and GC content was chosen for *T. palmi PFAS*.

The modified ASOs were delivered orally. Oral delivery of RNAi constructs by mixing with diet and feeding on dsRNA-treated leaf discs are effective in *Frankliniella occidentalis*, and *T. tabaci* [28,39,40]. The previously reported diets for thrips such as the LB diet [27] and the TSBY diet [28] were tried for *T. palmi* initially. However, these did not work well for *T. palmi*. In our previous study, pine pollen was found suitable for the fitness of *T. palmi* [26]. Therefore, ASO was delivered by mixing with an extract of pine pollen and sucrose. A blue tracker dye was mixed with the diet. The dye was visible through the thrips cuticle, helping ascertain the successful uptake of ASO by thrips. The microcentrifuge tube was modified as an artificial feeding setup for the delivery of ASO. Similar types of delivery setups were earlier used by Jahani et al. [41], Andongma et al. [28], and Zhang et al. [40] to deliver RNAi constructs in thrips. *T. palmi* adults and larvae survived in this setup for more than 12 days providing sufficient time for the assay. The same setup may also be useful to deliver or evaluate other RNAi constructs or anti-thrips molecules.

Based on the initial dose–response, ASOs were evaluated at 600 pmol/mL. Feeding of ASOs by *T. palmi* adults induced up to 2.70-fold downregulation in target mRNAs. The silencing of target mRNA was accomplished by RNAse H-mediated target-specific mRNA degradation and/or steric hindrance of ribosomal activity. However, the actual downregulation of target mRNA might be many times more than the estimated values. A large portion of thrips was killed post-exposure to ASO and their transcripts were not well represented in the estimation of target mRNA expression.

Although there was no immediate mortality in *T. palmi* adults with ASO treatment, ASO exposure negatively affected the fitness of *T. palmi* with a longer exposure. The delayed effect of ASO is common in insects. In larvae of *Unaspis euonymi* and *Dynaspidiotus britannicus*, the highest mortality and decrease in target mRNA expression was recorded at 10 days of ASO treatment [42]. A similar trend of the delayed effect of ASO on thrips mortality and mRNA expression was recorded in the present study. The silencing of *T. palmi UHRF1BP1* and *PFAS* induced up to 93.33% mortality in *T. palmi* adults at 4 DPA. The mortality was further increased up to 100% with longer exposure. The silencing of *V-ATPase-B*, *SNF7*, and *AQP* genes in *F. occidentalis*, and *T. tabaci* reported up to 29, 62, and 72% mortality [43,44]. The present study is the first to silence *UHRF1BP1* and *PFAS* of a thrips species and assess the effect on thrips survival.

*T. palmi* acquires GBNV during the early larval stage and adults can only transmit the virus. Thus, the effect of *T. palmi UHRF1BP1* and *PFAS* silencing on GBNV acquisition was evaluated at the larval stage. Exposure to ASO induced a reduction in GBNV copies acquired by *T. palmi* larvae. Silencing of *PFAS* has a greater effect on GBNV acquisition than *UHRF1BP1* silencing. This is the first report on the regulatory role of *UHRF1BP1* and *PFAS* of thrips in tospovirus infection. However, the GBNV copies increased with time and there was no significant difference in GBNV copies in adults post-exposure to ASOs during the L1 stage compared to the thrips fed on the diet without ASO. The expression of *UHRF1BP1* and *PFAS* mRNA in *T. palmi* adults that were exposed to ASO during the L1 stage was also on a par with *T. palmi* fed on the diet without ASO. *T. palmi* underwent four molts post-ASO exposure and became adults in 8–10 DPA. The efficacy of ASO might not persist either for a longer period or across the molts. This is especially true for the inhibition of virus replication [45]. In clinical trials, the ASO-based drugs are effective at once-weekly application [46]. However, the efficacy of ASO was reported for up to 30 days against *Candidatus* Liberibacter asiaticus (*C*Las) in citrus [47]. In the present study, the maximum efficacy of ASO was recorded at 8 DPA when applied to the adult stage.

The respective sense oligos were also fed to *T. palmi*. No corresponding effect of the sense oligos was recorded in *T. palmi*. Thus, the downregulation of target mRNA, mortality, and reduction in GBNV titer were specific to ASOs. The ASOs targeting thrips mRNA were also fed to *B. tabaci.* No specific effect of ASO was recorded in *B. tabaci* that indicated the specificity of ASOs to thrips and not to a non-target organism.

In conclusion, this is the first-ever report of silencing *T. palmi UHRF1BP1* and *PFAS* using ASO to induce mortality and impair virus transmission in *T. palmi*. *T. palmi UHRF1BP1* and *PFAS* would be novel genetic targets to manage thrips and restrict the spread of tospoviruses.

## Figures and Tables

**Table 1:** Antisense oligonucleotide sequences targeting *Thrips palmi UHRF1BP1* and *PFAS*.

S. N.	ASO Name	ASO Sequence (5′→3′)	Length (Nucleotide)	Gene Target	GC %	∆G (kcal/mol)	Unpaired Probability of Target Site Nucleotide
1	AG459-UHRF1BP1-ASO	*A*C*CATCC*T*GC*G*ACCCTTCT*G	20	*T. palmi* *UHRF1BP1*	55	−12.3	0.570
2	AG461-PFAS-ASO	*G*GATGCTGATACAGCCTTA*A	20	*T. palmi* *PFAS*	45	−8.9	0.563
3	UHRF1BP1 sense oligo	*C*AGAAGGGTCGCAGGATGG*T	20	*T. palmi* *UHRF1BP1*	60		
4	PFAS sense oligo	*T*TAAGGCTGTATCAGCATC*C	20	*T. palmi* *PFAS*	45		

Asterisk (*) indicates phosphorothioate modification by replacing the nonbridging oxygen atom with a sulfur atom to enhance nuclease stability.

**Table 2:** List of primers used in this study.

S.N.	Forward Primer	Forward Primer Sequence (5′→3′)	Reverse Primer	Reverse Primer Sequence (5′→3′)	Target Gene	Amplicon Length (bp)	Reference
1	AG109F	CCATCTACTTCAGTAGAAAACACTAG	AG110R	AGAGCAATCAGTGCAACAATTAAATA	GBNV M segment	1767	[14]
2	AG335F	CATCTGGCCCTACGTCAG	AG336R	CTGGTGGCTCTGCAGATG	GBNV nucleocapsid protein (N) gene	219	This study
3	AG171F	CCAGCCACATTCCTGGATAC	AG172R	ATGCGTTGGCAGTCACATAC	*T. palmi* *β-tubulin*	117	[13]
4	AG369F	CTGCGATGGATTCTGGGAAA	AG370R	GTCAGGCCAACTTTCTGACA	*T. palmi PFAS*	170	This study
5	AG379F	CGGAGGTGCTCTTCAAATCA	AG380R	AACTGCACGGTTGCTTTCTA	*T. palmi UHRF1BP1*	190	This study

## Data Availability

The datasets presented in this study can be found in online repositories. The names of the repository/repositories and accession number(s) can be found at: https://www.ncbi.nlm.nih.gov (accessed on 28 July 2021).

## Supplementary Materials

The following supporting information can be downloaded at: https://www.mdpi.com/article/10.3390/pathogens11111319/s1, Figure S1: Response of *Thrips palmi* to different doses of ASO; Figure S2: Effect of ASOs on the fitness of *Bemisia tabaci*.
