# Supplementary material for: Silencing of Thrips palmi UHRF1BP1 and PFAS Using Antisense Oligos Induces Mortality and Reduces Tospovirus Titer in Its Vector
Source: Pathogens. 2022 Nov 10;11(11):1319. doi: 10.3390/pathogens11111319 (PMC9695589; doi:10.3390/pathogens11111319)
Supplement: Supplementary file 1 [file pathogens-11-01319-s001.zip › pathogens-1936818-supplementary.pdf]

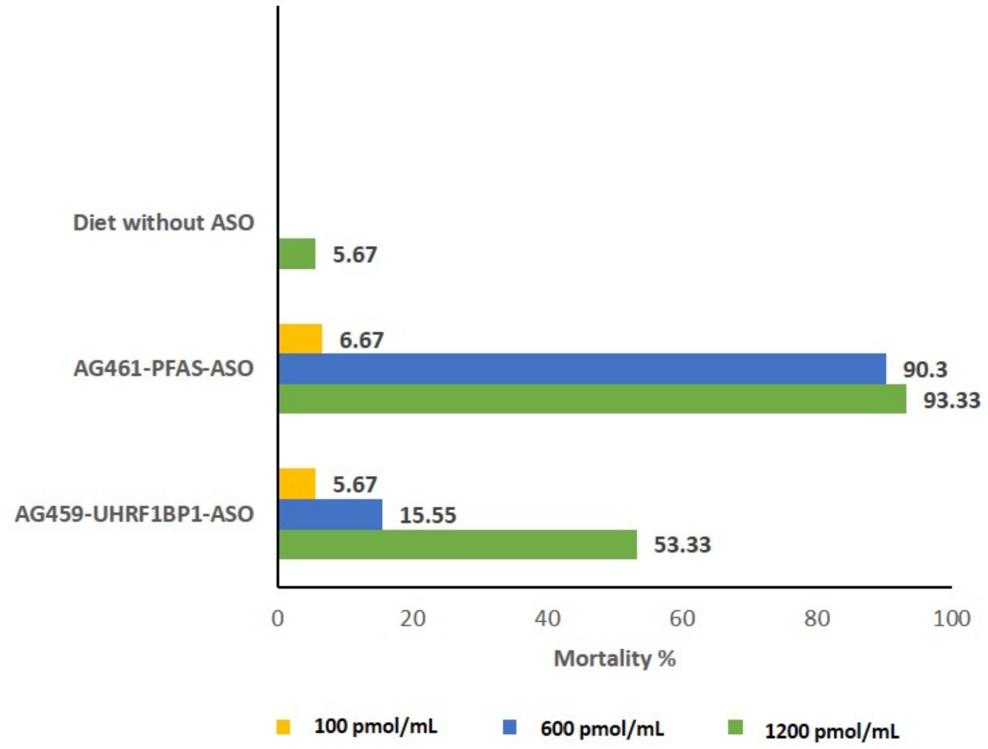

Supplementary Figure S1. Response of *Thrips palmi* to different doses of ASO.

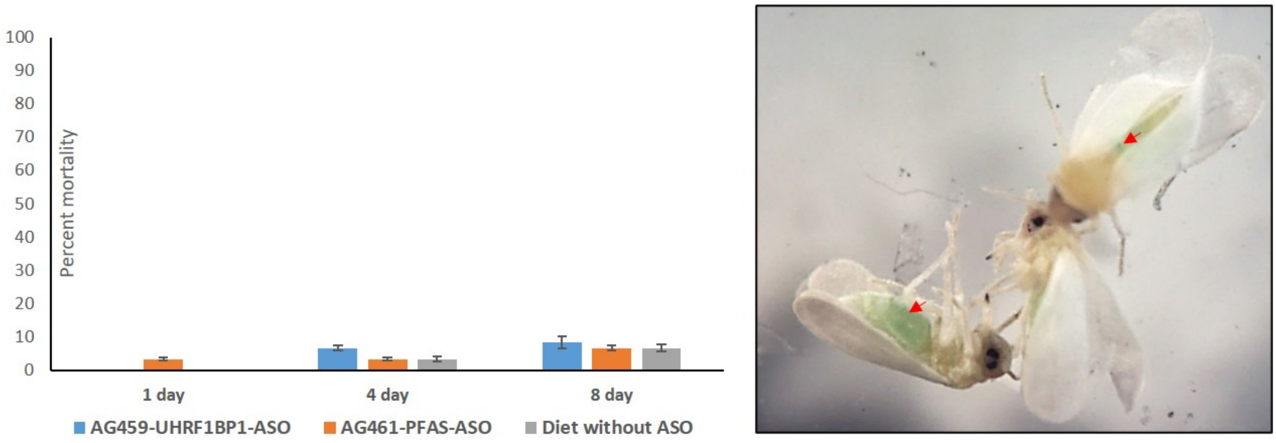

Supplementary Figure S2. Effect of ASOs on the fitness of *Bemisia tabaci*. The red arrow indicates a blue tinge in the abdomen due to feeding of ASO mixed diet.
